# Collision tumor of low-grade B-cell lymphoma and adenocarcinoma with tuberculosis in the colon: a case report and literature review

**DOI:** 10.1186/1477-7819-12-147

**Published:** 2014-05-11

**Authors:** Hung-Hsin Lin, Jeng-Kai Jiang, Jen-Kou Lin

**Affiliations:** 1 Division of Colon & Rectal Surgery, Department of Surgery, Taipei Veterans General Hospital, No 201, Sec. 2, Shih-Pai Road, Taipei 11217, Taiwan; 2 National Yang-Ming University, No.155, Sec.2, Linong Street, Taipei 11221, Taiwan

**Keywords:** Adenocarcinoma, Collision tumor, Colon, Lymphoma, Tuberculosis

## Abstract

This report presents a case of collision tumors of low-grade B-cell lymphoma and adenocarcinoma in the sigmoid colon of an 81-year-old man. All surgically resected regional mesenteric lymph nodes were found to be occupied by low-grade B-cell lymphoma, and one lymph node showed the presence of adenocarcinoma. Low-grade B-cell lymphoma was also observed in the resected spleen. Moreover, concurrent tuberculosis infection in the resected colon was proven by the presence of positive results obtained with polymerase chain reaction analysis of the mycobacterial DNA. Systemic chemotherapy was administered for advanced colon cancer with lung metastasis, and anti-tuberculosis treatment was also prescribed. The occurrence of synchronous lymphoma and adenocarcinoma of the colorectal region is rare. Furthermore, collisions of these different entities are also extremely unusual. The accurate clinical determination of the dominant tumor and a timely follow-up are required for the proper treatment of these cases.

## Background

Only a small number of studies on collision tumors of lymphoma and colorectal adenocarcinoma exist [1-8]. In non-Hodgkin’s lymphoma (NHL), the gastrointestinal tract is the most frequently involved extranodal site, with the stomach being the most common location (50% to 60%) followed by the small intestine (30%). Primary lymphoma of the colon is rare, comprising only 0.2% to 1.2% of all colonic malignancies [9]. Additionally, colon lymphomas represent 5.6% to 20% of all gastrointestinal lymphomas [10]. Furthermore, lymphoma synchronously accompanied by adenocarcinoma of the colon is extremely rare [6].

## Case presentation

**Figure 1:**
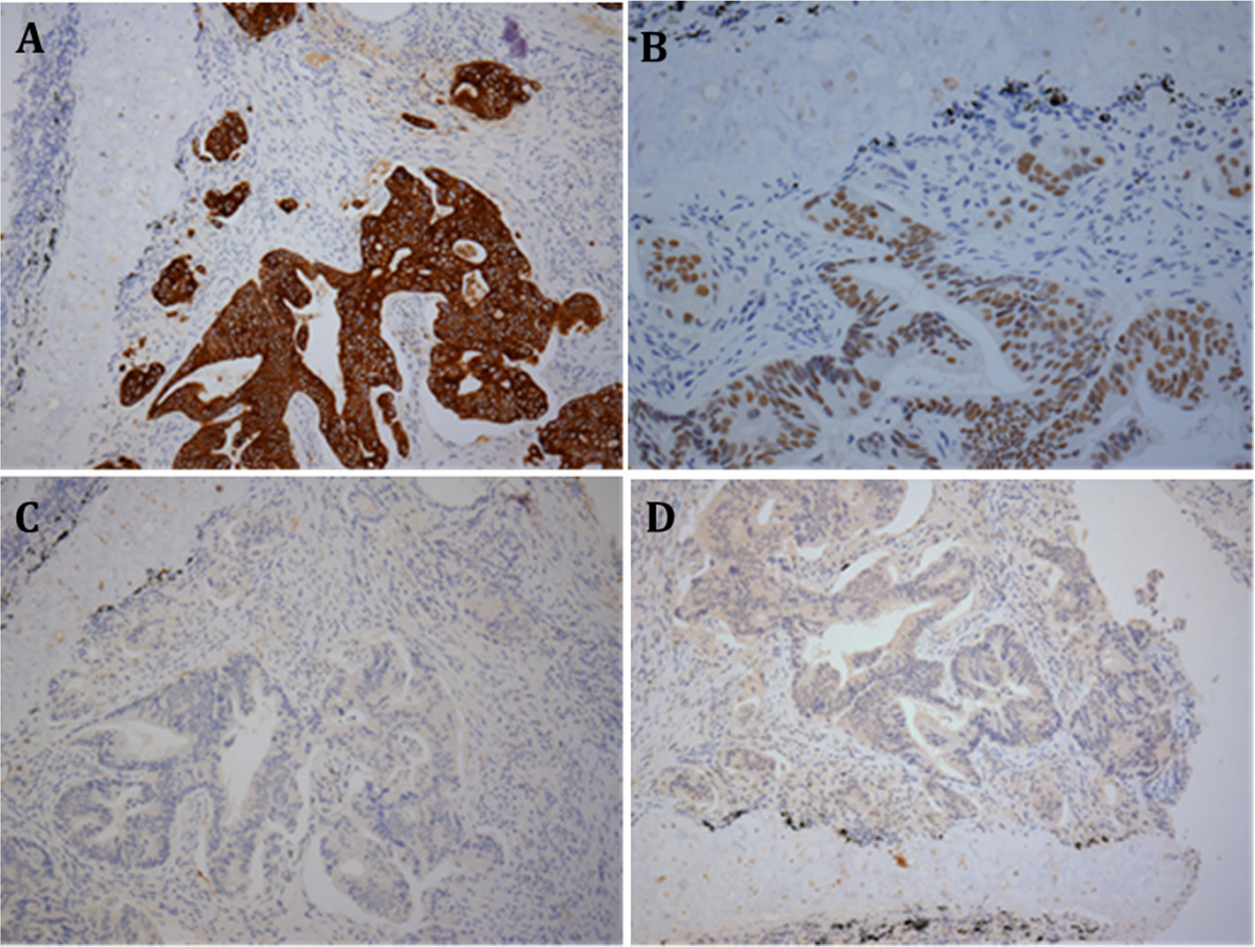
**Histological appearance of the lung biopsy specimen.** Malignant tumor cells were arranged in clusters and an acinar pattern, suggestive of adenocarcinoma. **(A)** Positive immunohistochemical staining for cytokeratin 20. **(B)** Positive immunohistochemical staining for caudal-related homeodomain transcription factor 2. **(C)** Negative immunohistochemical staining for cytokeratin 7. **(D)** Negative immunohistochemical staining for thyroid transcription factor-1.

**Figure 2:**
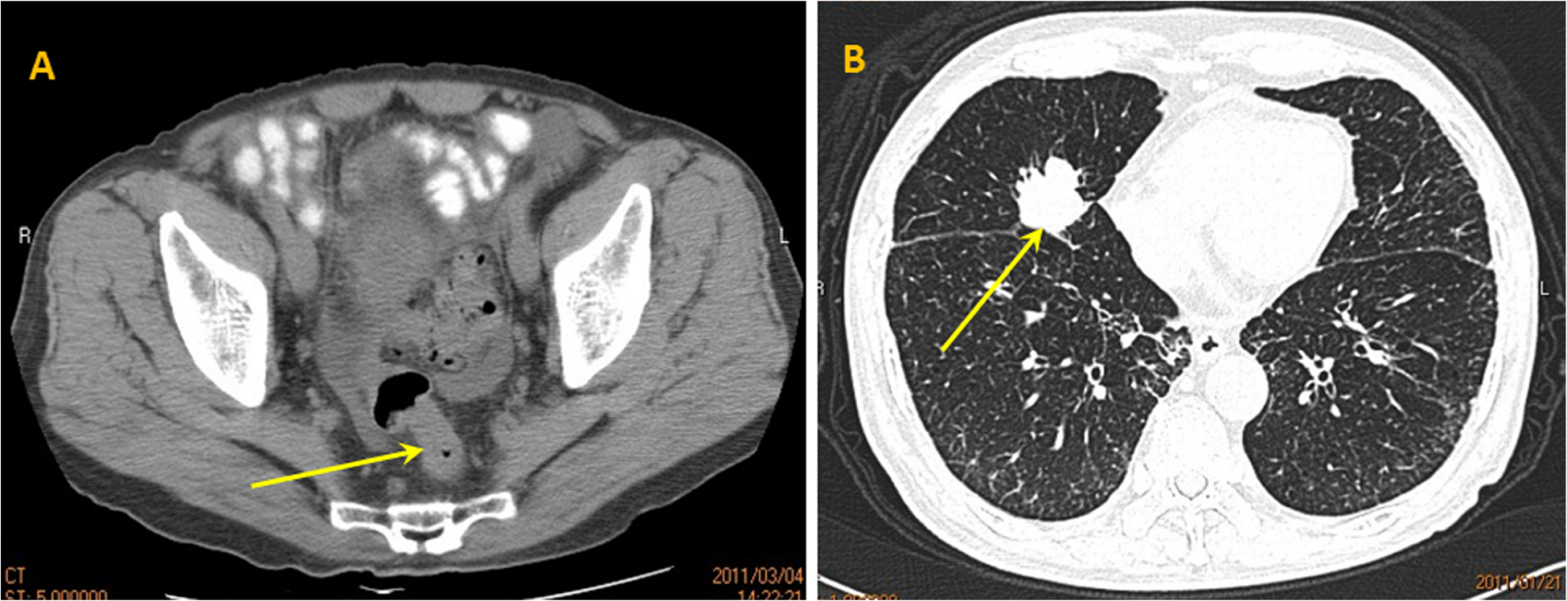
**Computed tomography (CT) of the abdomen and chest. (A)** Abdominal CT revealed wall thickening of the sigmoid colon, which is compatible with colon cancer (arrow). **(B)** Chest CT revealed a 3.2-cm nodule in the right middle lung field (arrow).

**Figure 3:**
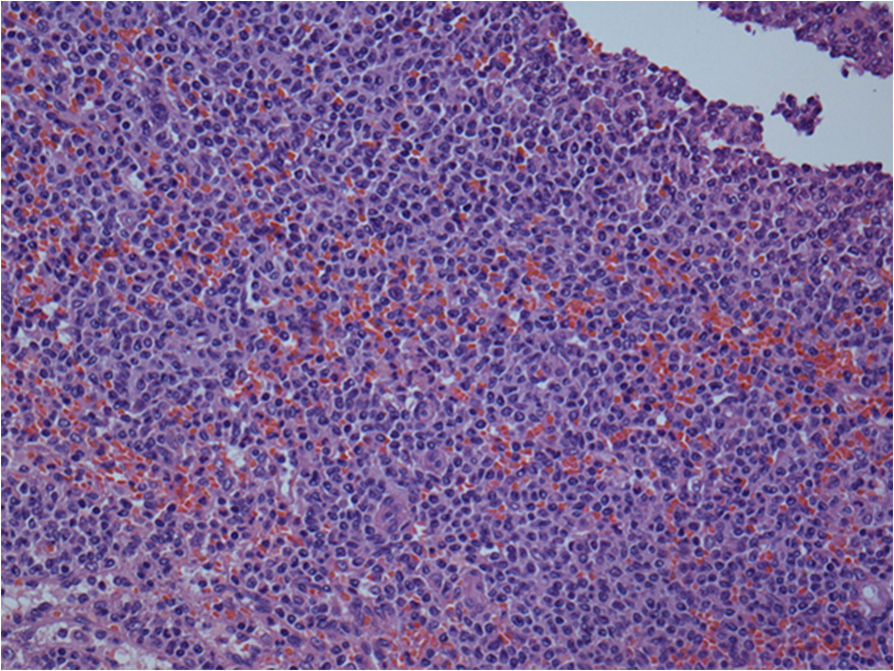
**The spleen was infiltrated by low-grade B-cell lymphoma with the proliferation of small-to-medium-sized cells.** Hematoxylin and eosin, original magnification, ×100.

An 81-year-old man presented to the Department of Chest Medicine as an outpatient with a chronic dry cough persisting for 1 month. There were no other systemic symptoms, excluding hypertension and benign prostate hyperplasia. Chest radiography revealed a 3.2-cm nodule in the right middle lung field. Bronchoscopic biopsy was performed, and the pathological examination revealed adenocarcinoma, which was considered to be colorectal in origin, as proved by a positive immunostaining reaction for cytokeratin 20 (Figure 1A) and caudal-related homeodomain transcription factor 2 (Figure 1B) as well as immunostaining reaction for cytokeratin 7 (Figure 1C) and thyroid transcription factor-1 (Figure 1D). The patient was then referred to the outpatient Department of Colorectal Surgery. After admission, a series of analyses were performed. The general examination was unremarkable, with no lymphadenopathy, and laboratory studies revealed a hemoglobin concentration of 11.9 g/dL, a white blood cell count of 5,800 cells/μL, and a platelet count of 154,000 cells/μL. Renal and liver function tests were normal, and hepatitis markers were negative. Serum carcinoembryonic antigen and carbohydrate antigen 19-9 levels were 214 ng/mL and 1.21 U/mL, respectively. A complete colonoscopy revealed an annular ulcerative lesion in the sigmoid colon (25 cm above the anal verge). Biopsy of the tumor was performed, which revealed a moderately differentiated colon adenocarcinoma. Computed tomography of the abdomen and pelvis revealed mesenteric lymphadenopathies associated with a mass in the sigmoid colon (Figure 2A, arrow). There was no liver metastasis. Computed tomography of the thorax revealed a 3.2-cm nodule in the right middle lung field that was consistent with lung metastasis (Figure 2B, arrow). The patient underwent anterior resection associated with regional lymphadenectomy, with the pathological assessment of the resected specimen revealing a collision tumor consisting of a moderately differentiated adenocarcinoma extending through the muscularis propria and low-grade B-cell lymphoma. Extramural vascular invasion of the adenocarcinoma cells was present. Interestingly, there were several granulomatous lesions on the resected sigmoid colon. Acid-fast staining and polymerase chain reaction analysis of mycobacterial DNA revealed positive findings. Microscopic evaluation of the 19 regional lymph nodes in the mesentery of the resected colon showed diffuse infiltration of low-grade B-cell lymphoma in the lymph node architecture. In one lymph node, metastasis of adenocarcinoma was also present. According to immunohistochemistry, the cells were positive for cluster of differentiation (CD) 20, B-cell lymphoma (Bcl)-2, and CD10 (weak), and negative for CD3, CD5, CD23, Bcl-6, terminal deoxynucleotidyl transferase, and cyclin D1. The proliferation fraction (MIB-1 immunostaining) was approximately 15%. The morphological and immunohistochemical findings were used to confirm the diagnosis of synchronous presentation of low-grade B-cell lymphoma and colon adenocarcinoma within the sigmoid colon tumor and mesenteric lymph nodes. Furthermore, microscopic examination of the spleen revealed infiltration of the specimen by low-grade B-cell lymphoma with proliferation of small to medium-sized cells (Figure 3). *K-RAS* and *B-RAF* genetic mutations were evaluated in the primary tumor DNA after microdissection, and both genes were determined to be the wild type. Three weeks after surgery, the patient was scheduled to undergo a bone marrow biopsy for lymphoma staging, but he declined because of his advanced age and general poor condition.

After discussion of this case in a multidisciplinary team treatment conference for colorectal cancer, the patient underwent palliative chemotherapy with modified fluorouracil, leucovorin, and oxaliplatin (mFOLFOX6) for six cycles. However, he presented with grade III peripheral neuropathy in both hands and feet. On the basis of the late stage of the bowel malignancy, advanced age, and complexity of comorbidities of the patient, we proposed a palliative treatment with capecitabine, which is ongoing. He also received tuberculosis (TB) treatment, which included ethambutol, rifampicin, isoniazid, and pyrazinamide for the first 2 months, and then ethambutol, rifampicin, and isoniazid for the next 6 months, after extra-pulmonary TB was detected. He was regularly followed-up as an outpatient. The patient has remained in a stable condition for 24 months after surgical resection of the colon.

## Discussion

To our knowledge, this report is the first case of a collision tumor of low-grade B-cell lymphoma and adenocarcinoma in the same colonic segment, and retrieved lymph nodes coexisting with a TB infection.

The coexistence of TB and carcinoma is well documented in the lungs, skin, and larynx. However, the simultaneous occurrence of these two diseases is rarely observed in the colon. Chronic diseases, such as ulcerative colitis and Crohn’s disease, are known to increase the risk of malignancy, but the causal relationship between carcinoma and TB in the colon is still unclear [11]. The etiological relationship between the two diseases is a matter of debate. The coexistence of TB and adenocarcinoma in the colon may be coincidental, or one disease process might have initiated the other [12-14]. Falagas et al. [15] reported that clinicians need to be aware of the protean manifestations of TB and cancer, and they should maintain a high index of suspicion for simultaneous and/or misleading presentations. TB and various types of malignancies can mimic each other and have atypical clinical and radiological expressions. Further research is required to determine whether TB infection, being similar to other chronic infections and inflammatory conditions, may facilitate carcinogenesis.

Molecular genetic analysis is of special importance for the diagnosis of collision tumors consisting of poorly differentiated neoplasms, such as peripheral T-cell lymphoma and anaplastic carcinoma, particularly when the results of immunohistochemistry are inconclusive. We believe that collision tumors consisting of large cell NHL and carcinoma would be detected more often if molecular genetic analysis were used more extensively [4]. Although we were unable to identify any common etiologic factor of the two malignancies in our patient, we believe that their synchronous presentation is purely coincidental. Nevertheless, in addition to its uniqueness, the situation of this patient offers several therapeutic challenges for clinicians. The advanced age of the patient, metastatic colorectal cancer, the influence of one malignancy on the natural history of another, and extra-pulmonary TB increased the complexity of treatment.

There are some hypotheses suggesting the etiology of collision tumors. One hypothesis is that the two primary tumors arise in continuity through a chance accidental ‘meeting’. On the contrary, to date, there are no known common etiologic factors for the occurrence of both adenocarcinoma and malignant lymphoma in the colon. Another hypothesis is that the presence of the first tumor alters the microenvironment, precipitating the development of the second adjacent tumor [7].

In our case, the pre-operative examinations were unable to reveal lymphoma adjacent to adenocarcinoma. Further intensive investigations may help to detect the existence of collision tumors. Herein, we reported a rare case of collision tumors (adenocarcinoma and low-grade B-cell lymphoma) in the colon. A therapeutic dilemma exists in deciding the course of treatment in patients with collision tumors. Collision tumors of two types, similar to the current case, were previously observed in 8 patients, as listed in Table 1. The age of the patients ranged from 59 to 81 years, with a mean age of 68.9 years. The male to female ratio was 3:1. Adenocarcinomas and lymphomas were located in the right colon, left colon, and rectum in 5, 1, and 3 patients, respectively. Previous reports have been limited because of the rarity of such collision tumors, and most reports did not describe the course of the disease after diagnosis. Sasaki et al. [6] reported that a patient received six courses of combined chemotherapy (cyclophosphamide, doxorubicin, vincristine, and prednisone regimen with rituximab) against malignant lymphoma, which yielded a complete response. In our case, the patient underwent six courses of chemotherapy with mFOLFOX6 followed by capecitabine to treat sigmoid colon adenocarcinoma with lung metastasis. Takashi et al. [7] reported a patient who died because of the peritoneal recurrence of malignant lymphoma.

**Table 1:** Cases of collision tumors of primary malignant lymphoma and adenocarcinoma in the colon reported

**Source**	**Year**	**Age (years)/Sex**	**Tumor location**	**Tumor type**	**Pathological stage**	**Treatment**	**Prognosis**
Cornes et al. [1]	1960	63/M	Rectum	Lymphocytic lymphosarcoma	pT_3_N_0_M_0_	NA	NA
Aitani et al. [2]	1987	59/M	Transverse colon	Diffuse large B-cell lymphoma	NA	NA	Alive
Chazouillères et al. [3]	1990	56/F	Rectum	Lymphoma	NA	NA	NA
Mannweiler et al. [4]	2003	73/M	Rectum	T-cell lymphoma	pT_2_N_0_M_0_	NA	NA
Minato et al. [5]	2004	80/M	Ascending colon	Diffuse large B-cell lymphoma	NA	NA	5 months, died of pneumonia
Sasaki et al. [6]	2010	62/M	Cecum	Follicular lymphoma	pT_3_N_0_M_0_	CHOP + Rituximab	Alive
Takashi et al. [7]	2011	76/F	Ileocecal	Diffuse large B-cell lymphoma	pT_3_N_1_M_0_	NA	5 months, died of lymphoma
Chang et al. [8]	2011	NA	Ileocecal	Diffuse large B-cell lymphoma	NA	NA	NA
Our case	2014	81/M	Sigmoid colon	Low-grade B-cell lymphoma	pT_3_N_1a_M_1_	mFOLFOX6	Alive

NA, Not available; CHOP, Cyclophosphamide, doxorubicin, vincristine, and prednisone; mFOLFOX, Modified fluorouracil, leucovorin, and oxaliplatin.

## Conclusions

This clinical course clearly suggested the difficulty of selecting the optimal therapy for collision tumors of different malignant entities, and the importance of close follow-up for both lesions. More cases should be reported in the literature to define the principle management in such challenging scenarios. Moreover, pathologists should be aware of the existence of collision tumors in the small and large intestines. The appropriate selection of immunohistochemical tests may help to establish the diagnosis. Immunohistochemical results can be resolved by molecular analysis, particularly when lymphomas are components of collision tumors of the colon.

## Consent

Written informed consent was obtained from the patient for publication of this case report and the accompanying images. A copy of the written consent is available for review by the Editor-in-Chief of this journal.

## Abbreviations

Bcl: B-cell lymphoma; CD: Cluster of differentiation; mFOLFOX: Modified fluorouracil, leucovorin, and oxaliplatin; NHL: Non-Hodgkin’s lymphoma; TB: Tuberculosis.

## Competing interests

The authors declare that they have no competing interests.

## Authors’ contributions

All authors participated in the conception and design of the study. HL performed the clinical analysis and drafted the manuscript. JL performed surgery and supervised the study. HL, JJ, and JL were involved in the final editing. All authors read and approved the final manuscript.
